# Supplementary material for: Memory-induced alignment of colloidal dumbbells
Source: Sci Rep. 2023 Oct 13;13:17409. doi: 10.1038/s41598-023-44547-z (PMC10575873; doi:10.1038/s41598-023-44547-z)
Supplement: Supplementary file 1 — Supplementary Information 1. [file 41598_2023_44547_MOESM1_ESM.pdf]

Supplemental material: Memory-induced alignment of colloidal dumbbells

Karthika Krishna Kumar,[1] Juliana Caspers,[2] Félix Ginot,[1] Matthias Krüger,[2] and Clemens Bechinger[1]

[1] *Fachbereich Physik, Universität Konstanz, 78457 Konstanz, Germany*

[2] *Institute for Theoretical Physics, Georg-August Universität Göttingen, 37073 Göttingen, Germany*

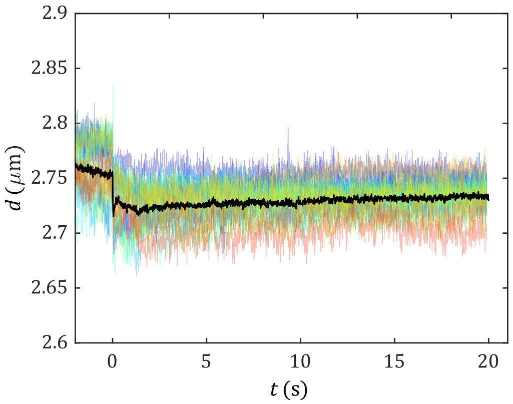

FIG. 1. Time evolution of distances between the dumbbell particles during a recoil for individual recoil runs (colored lines) and the mean distance (thick black line) for $v = 0.3\,\mu$m/s and $t_{\rm sh} = 50\,$s.

Interaction between dumbbell particles

Due to the small surfactant molecules in the solution, two particles when brought close to each other develop an attractive depletion interaction between them. These interactions are strong enough to keep the particles as a dumbbell during the recoil. Fig. 1 shows the distances, $d = \sqrt{(x_2 - x_1)^2 + (y_2 - y_1)^2}$, between the dumbbell particles with coordinates (x_1, y_1) and (x_2, y_2) during a recoil. The distance between the particles is larger during shear because the beam is deflected such that the two traps are not close enough for the particles to fall into the same trap.

Supplementary video 1

The video demonstrates a typical experiment where a dumbbell is dragged using an extended optical trap (green circles) and released to exhibit recoil motion. Since the optical trap and camera are stationary and the sample is translated using a piezo stage, the drag on the dumbbells cannot be visualized in the recorded experimental videos. For better visualization, we have shown the process from the coordinate system corresponding to the resting sample. This has been achieved by adding the translational stage velocity to the dumbbell's motion during the shear phase (corresponding to the first two seconds of the shown video). The dashed lines indicate the orientation of the dumbbell before release which is clearly different from the orientation towards the end of the recoil due to the MIA.

Decrease of initial angle during shear

The decrease in orientation angle can also be observed during shear (see Fig. 2). During shear, the dumbbell gradually tries to align with the shear direction and reaches a steady state orientation which corresponds to the θ_0 we measure.

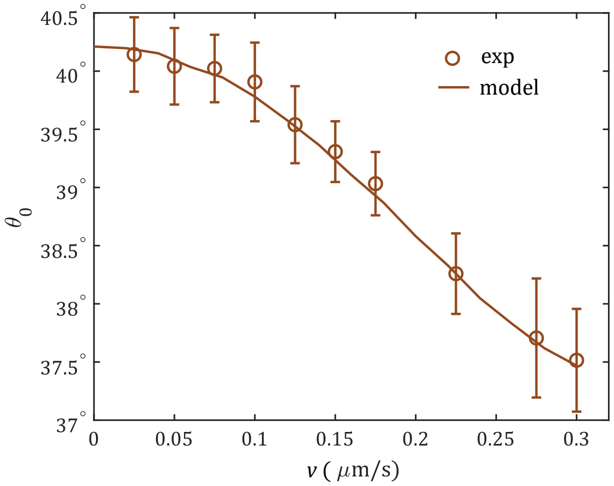

FIG. 2. Initial angle θ_0 measured 1 s before release for different shear velocities v (orientation in the static trap, $\theta_0^* = 40.2^\circ$ and $t_{\rm sh} = 50\,$s) for experiments (open symbols) and simulations (line).

Translational recoil under variation of initial angle

Fig. 3 shows the amplitude of translational recoil for different initial angle θ_0 in simulations (line) and experiments (symbols). In contrast to the MIA which shows a non-monotonic trend with θ_0, the amplitude of translational recoil doesn't show a significant dependence on the initial angle θ_0. Moreover, the error bars from experiments don't change with θ_0 either, highlighting that a frustrated state occurs only for the MIA and not for the translational recoil amplitudes. In the case of simulations, error bars are negligible and there has been no dependence of the variance on θ_0 observed.

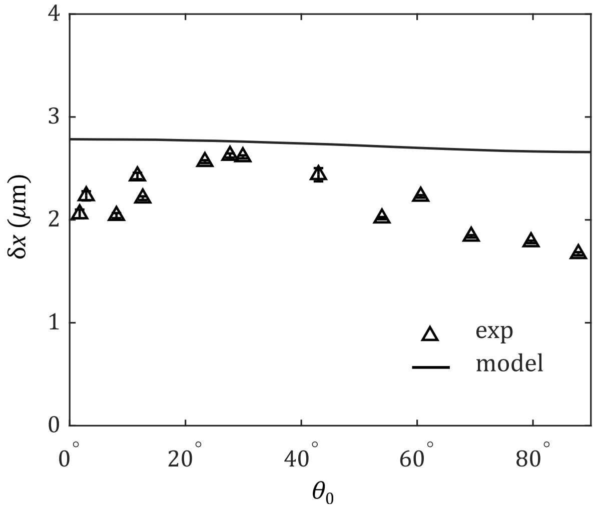

FIG. 3. Translational recoil amplitude for the variation of initial angle θ_0 for experiments (symbols) and simulations (line) for fixed shear velocity $v_{\rm sh} = 0.2\,\mu$m/s and $t_{\rm sh} = 50$ s. Experimental error bars show no dependence on θ_0; error bars from simulations are negligible.

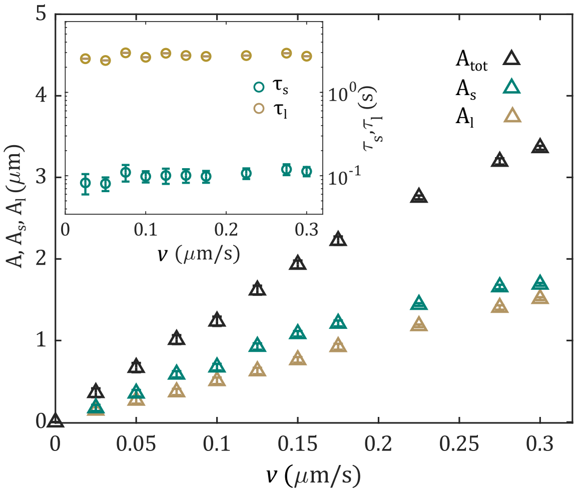

FIG. 4. Translational recoil amplitudes A_s, A_l and their sum A (main graph), together with respective amplitudes τ_s and τ_l (inset) obtained from fitting recoils for different shear velocities v ($\theta_0^* \sim 40^\circ$ and $t_{\rm sh} = 50$ s) to a double exponential form.

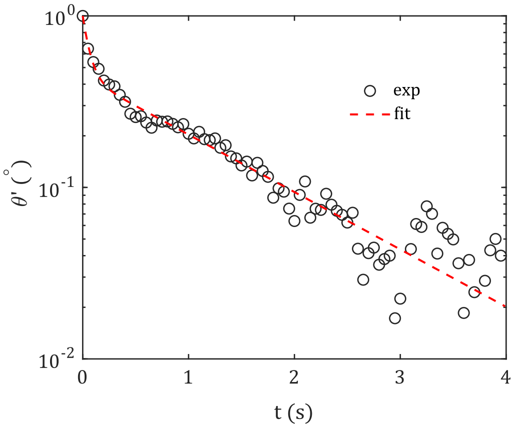

FIG. 5. Logarithmic plot of a normalized MIA curve $\theta' = 1 - (\theta_0 - \theta)/\delta\theta$ for $v = 0.3\,\mu$m/s, $t_{\rm sh} = 50$ s and $\theta_0 \sim 40^\circ$ with a double-exponential fit to Eq. (2) (dashed line).

Temporal evolution of translational recoil and MIA

The translational recoil is fitted well with a double-exponential form,

$$\delta x(t) = A - A_s e^{-t/\tau_s} - A_l e^{-t/\tau_l}, \qquad (1)$$

with short and long timescale, τ_s and τ_l, and respective amplitudes A_s and A_l, as well as their sum A. This agrees with previous findings for spherical probes [1, 2]. For the variation of shear velocity the resulting amplitudes and timescales after fitting experimental data to Eq. (1) are shown in Fig. 4. We find that all amplitudes scale linear with v, while the timescales are independent of shear velocity.

The MIA can be fitted to a double-exponential as well,

$$\delta\theta(t) = A^{(o)} - A_s^{(o)} e^{-t/\tau_s^{(o)}} - A_l^{(o)} e^{-t/\tau_l^{(o)}}, \qquad (2)$$

with timescales $\tau_s^{(o)}$, $\tau_l^{(o)}$ and respective amplitudes $A^{(o)}, A_s^{(o)}$ and $A_l^{(o)}$. However, due to bigger fluctuations compared to translational recoil, clear fit results are only found for the highest shear velocities. The experimental MIA curve (open symbols) for $v = 0.3\,\mu$m/s, together with the fit (dashed line) is shown in Fig. 5. The fit results are $A_s^{(o)} = 4.32^\circ$, $A_l^{(o)} = 3.38^\circ$, $\tau_s^{(o)} = 0.065$ s and $\tau_l^{(o)} = 1.29$ s.

Fitting the model to experiments

To find suitable model parameters that fit all experimental recoil amplitudes we follow a clear procedure: First we consider the angular and translational equilibrium mean squared displacements (MSAD, MSD) in Fig. 6 which determine the linear part of our model. At very short times, the two curves scale linearly with time (short-time diffusion), while they exhibit a sub-diffusive plateau [2–4] at intermediate times. At very long times the scaling becomes again linear and we observe long-time diffusion. Fitting the translational experimental equilibrium mean squared displacement MSD$(t) \equiv \langle (x(t) - x(0))^2 \rangle_{\rm eq}$ determines the translational friction coefficients γ and γ_b, and the harmonic part of the coupling between the two rods κ_2. We find $\gamma = 0.84\,\mu$Ns/m and $\gamma_b = 40\,\gamma$ from fitting short- ($\sim 2D_0 t$) and long-time diffusion ($\sim 2D_\infty t$), with $D_0 = k_B T/\gamma$ and $D_\infty = k_B T/(\gamma + \gamma_b)$. $\kappa_2 = 1.3\,\gamma$/s is determined by the plateau.

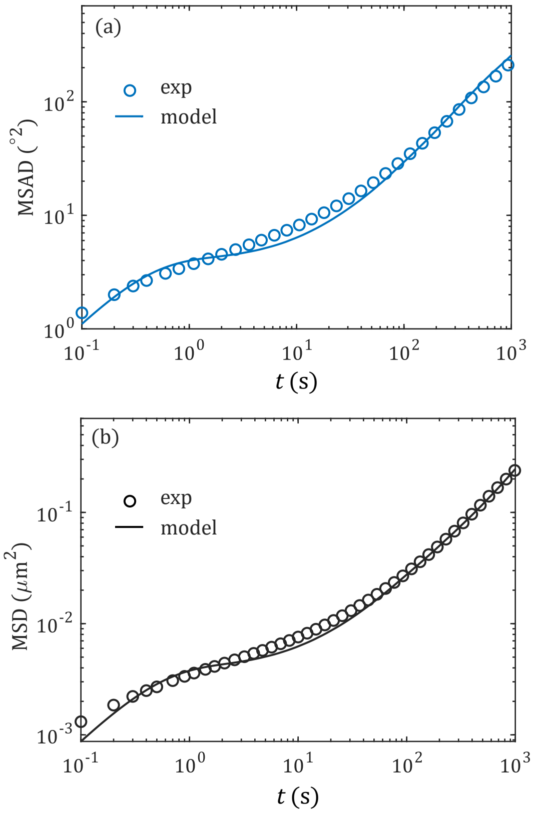

FIG. 6. (a) Angular and (b) translational equilibrium mean squared displacements (MSAD, MSD) for freely diffusing dumbbell particles in experiments (symbols) and simulations (lines).

The resulting simulated MSD in comparison to the experimental one is shown in Fig. 6(b) and shows good agreement.

Next, we consider the angular experimental equilibrium mean squared displacement $\mathrm{MSAD}(t) \equiv \langle(\theta(t) - \theta(0))^2\rangle_{\mathrm{eq}}$, see Fig. 6(a). This determines all rotational friction coefficients (γ_θ and $\gamma_{b,\theta}$) and the lengths of the probe (l) and bath rod (l_b). Note that the effective rotational coupling strength between probe and bath rod scales to lowest order $\propto \kappa_2 l l_b$ [5]; the nonlinearities of our model, κ_4 and κ_6, have negligible influence on MSAD. Comparing again short- and long-time diffusion (for γ_θ, $\gamma_{b,\theta}$) and the plateau (for l, l_b) we obtain $\gamma_\theta = 2.5\,\mu\mathrm{m}^2\gamma$, $\gamma_{b,\theta} = 127\,\mu\mathrm{m}^2\gamma$, $l = 2.1\,\mu$m and $l_b = 6\,\mu$m. The resulting simulated curve is shown as line in Fig. 6(a). To obtain the missing nonlinear coupling strength κ_4 we fit the amplitude of MIA, e.g. under variation of shear velocity. This yields $\kappa_4 = -7.15 \times 10^{-3}\,\gamma/\mathrm{s}$. For κ_6 we choose $\kappa_6 = 1.48 \times 10^{-5}\,\gamma/\mathrm{s}$ to have no influence in the regime which we probe. It is needed to obtain a well-defined steady state.

Next, we consider the variation of shear time which probes the regime before a steady-state has been reached, and therefore allows insight into the confinement strength during shear. Comparing MIA and translational recoil amplitudes, we can adjust the confinement strength of COM during shear to find $\kappa_x = 3.3\,\gamma/\mathrm{s}$ (see Fig. 5 in the main text). Finally, we compare the decrease of θ_0 during shear (see Fig. 2) from which we obtain the strength of orientation confinement during shear. We find $\kappa_\theta = 4.1\,\mu\mathrm{m}^2\gamma/\mathrm{s}$ for the variation of velocity and initial angle, and $\kappa_\theta = 6\,\mu\mathrm{m}^2\gamma/\mathrm{s}$ for the variation of shear time. Note that for Fig. 6(a) in the main text we set $\kappa_\theta = 30\,\mu\mathrm{m}^2\gamma/\mathrm{s}$ to approximately fit the experimentally observed orientational variance during shear.

TABLE I. Model parameters used in simulations.

γ	$0.84\,\mu$Ns/m
γ_b	$40\,\gamma$
γ_θ	$2.5\,\mu\mathrm{m}^2\gamma$
$\gamma_{b,\theta}$	$127\,\mu\mathrm{m}^2\gamma$
l	$2.1\,\mu$m
l_b	$6\,\mu$m
κ_2	$1.3\,\gamma/\mathrm{s}$
κ_4	$7.15 \times 10^{-3}\,\gamma/\mathrm{s}$
κ_6	$1.48 \times 10^{-5}\,\gamma/\mathrm{s}$
κ_x	$3.3\,\gamma/\mathrm{s}$

[1] F. Ginot, J. Caspers, L. F. Reinalter, K. K. Kumar, M. Krüger, and C. Bechinger, New J. Phys. **24**, 123013 (2022).
[2] J. Caspers, N. Ditz, K. Krishna Kumar, F. Ginot, C. Bechinger, M. Fuchs, and M. Krüger, J. Chem. Phys. **158**, 024901 (2023).
[3] J.-H. Jeon, N. Leijnse, L. B. Oddershede, and R. Metzler, New J. Phys. **15**, 045011 (2013).
[4] M. Weiss, Phys. Rev. E **88**, 010101 (2013).
[5] This can be found from an expansion of the torque acting on the probe to linear order in $(\theta - \theta_b)$, and assuming zero distance between the center of masses of probe and bath rod.
